# The effect of isoflurane anaesthesia and buprenorphine on the mouse grimace scale and behaviour in CBA and DBA/2 mice

**DOI:** 10.1016/j.applanim.2015.08.038

**Published:** 2015-11

**Authors:** Amy Miller, Gemma Kitson, Benjamin Skalkoyannis, Matthew Leach

**Affiliations:** School of Agriculture, Food and Rural Development, Agriculture Building, Newcastle University, Newcastle upon Tyne NE1 7RU, United Kingdom

**Keywords:** Mouse, Mouse grimace scale, Isoflurane, Buprenorphine

## Abstract

Prevention or alleviation of pain in laboratory mice is a fundamental requirement of *in vivo* research. The mouse grimace scale (MGS) has the potential to be an effective and rapid means of assessing pain and analgesic efficacy in laboratory mice. Preliminary studies have demonstrated its potential utility for assessing pain in mouse models that involve potentially painful procedures. The next step in validation is to determine if the other procedures that are integral to these models, i.e. anaesthesia or analgesia, result in any changes in MGS score which would need to be taken into account when using this tool to assess post-procedural pain. Here, spontaneous behaviour and MGS data for CBA and DBA/2 mice were recorded at baseline and following either isoflurane anaesthesia (suitable to perform abdominal surgery) or 0.05 mg/kg s.c. buprenorphine. In line with previous studies, isoflurane anaesthesia alone had limited effects on the spontaneous behaviour in either strain of mice. Administration of buprenorphine resulted in increased periods of activity e.g. walking and chewing bedding in CBA mice. These effects were not demonstrated in DBA/2 mice. In comparison, buprenorphine alone had no impact on MGS score in either strain of mice, however DBA/2 mice showed a significant increase in MGS score following isoflurane anaesthesia. The presence of this increased MGS score must be taken into account when attempting to use the MGS to assess pain in DBA/2 mice. Further work should be carried out to establish the presence of this isoflurane effect in other strains and the potential influence of gender on the MGS. This further validation is necessary prior to implementation of this technique in clinical scenarios.

## Highlights

• Isoflurane anaesthesia increases MGS score in male DBA/2 mice compared to baseline.
• Isoflurane anaesthesia alone has no effect on mouse grimace scale score in male CBA mice.
• 0.05 mg/kg buprenorphine has no effect on MGS score in either male CBA or DBA/2 mice.

## 1 Introduction

Prevention or alleviation of pain in laboratory animals is a fundamental requirement of *in vivo* research. This is particularly critical for laboratory mice, as they comprise the greatest number of animals used, and this number is increasing annually with the growth of research using genetically modified models (Home Office, 2012). With this increasing diversity of mouse strains and models in common use, we need to ensure that our means of assessing pain and analgesic efficacy are both effective and clinically applicable across mouse strains. To date, many of the studies carried out to develop and validate indices of pain in mice have not looked at effect of strain. In those that have strain has been shown to have a significant effect on behaviour, responses to routine anti-nociceptive testing, and analgesia and anaesthesia efficacy (Kim et al., 2005, Mogil et al., 1999, Kest et al., 2002, Groeben et al., 2003, Dickinson et al., 2009).

In order for an index of pain to be applied clinically we need to establish its validity by ensuring the measure relates directly to pain, that it can be easily and rapidly carried out with a minimum of interference to both the animals and care staff and that it can be measured accurately with little variation between or within observers. Recently, spontaneous ‘pain’ behaviour and facial expressions have been investigated as a means of effectively assessing pain in laboratory mice (e.g. Leach et al., 2012, Miller et al., 2012, Wright-Williams et al., 2013).

Following vasectomy, key changes in behaviour have been identified in male CD1 mice (Miller et al., 2012, Leach et al., 2012). These include decrease in the presence of normal exploratory behaviours including rearing from pre to post-op and the presence of abnormal, ‘pain’ behaviours including twitching and writhing, post-op which are not presented under baseline conditions. These changes in behaviour are reduced by administration of an analgesic but not prevented (e.g. Leach et al., 2012). Manual scoring of mouse behaviour is however extremely time consuming, limiting the number of strains of mice, procedures and analgesics that can be screened for effectiveness.

The mouse grimace scale (MGS) devised by Langford et al. (2010) is being considered as a potentially accurate and reliable means of scoring pain in mice. Langford et al. (2010) demonstrated an increase in MGS score following a range of potentially painful procedures including laparotomy. In a further study MGS was shown to be potentially valid for assessing pain associated with scrotal approach vasectomy in CD1 mice (Leach et al., 2012), where a significant increase in MGS score was observed from pre to post surgery. This effect was reduced by the administration of an analgesic. An identical pattern was also observed when manually scoring key, previously identified, pain associated behaviours in the same mice.

When validating these ‘pain associated’ behaviours and facial expressions for assessing pain under clinical conditions, we need to ensure that the other non-painful procedures that are integral to the research being carried out (e.g. anaesthesia or analgesia) are either minimal or that the magnitude of the effect is understood and can be taken into account when assessing mice. Although, previous studies have demonstrated that procedures such as administration of buprenorphine can causes behavioural changes in mice such as alterations in activity levels (Cowan et al., 1977, Tubbs et al., 2011, Wright-Williams et al., 2013), to date the effect of the majority of routinely used analgesics or anaesthetics on the MGS in animals not in pain has not been assessed.

Following a surgical procedure, mice are often not administered analgesia (Richardson and Flecknell, 2005, Stokes et al., 2009). When analgesia is provided to laboratory mice, it is given in a range of scenarios, which do not all involve general anaesthesia. It was therefore decided to study the effects of isoflurane and buprenorphine separately to provide data that is relevant in a range of clinical scenarios. Here, we aim to study two common strains of laboratory mice, CBA and DBA/2, to determine if the administration of anaesthetic (isoflurane) or an analgesic (buprenorphine) alone results in changes in MGS score or spontaneous behaviour.

## 2 Materials and methods

All procedures were conducted in accordance with the Animals (Scientific Procedures) Act 1986, European Directive EU 2010/63 and with the approval of Newcastle University Animal Welfare and Ethics Board.

### 2.1 Animals

Eight CBA and Eight DBA/2 male mice (Charles River Laboratories Inc, Kent) weighing 25.6–28.7 g (CBA) and 23.3–26.3 g (DBA/2) at the start of the study were used. Mice were housed in same strain groups of 4 in individually ventilated cages (IVCs) (Type 2 – Arrowmight, Hereford, UK) with sawdust bedding and nesting material (sizzle nest, Datesand Ltd, Manchester, UK). Environmental enrichment was provided in the form of chew blocks and cardboard tubes (Datesand Ltd, Manchester, UK). A seven-day acclimation period was given prior to the start of the study. The animal room was maintained at 23 °C ± 1 °C, 48% humidity and on a 12/12 h light dark cycle (lights on at 07:00). Food CRM (P); SDS Ltd, Essex, UK and tap water were provided *ad libitum*.

### 2.2 Sample size

A sample size calculation was carried out using G*Power (V.3.1.) using data from Leach et al. (2012) and assumed power of 80%. Calculations indicated a minimum sample size *n* = 6.

### 2.3 Data collection

#### 2.3.1 Baseline data collection

Mice were placed individually into small custom made chambers (80 × 80 × 80 mm^3^) for a six minute habituation period. Following this, close up, high definition (HD) images of their faces were recorded during a 3 min session. Mice were then immediately placed individually in clear plastic cages (350 × 200 × 140 mm^3^) (Techniplast UK Ltd, London, UK) that contained only bedding. The behaviour of each individual was recorded, in HD for 10 min using a video camera (HDR-XR155, Sony, Japan) positioned at a fixed distance from the cage. Following filming the mice were returned to their home cages.

#### 2.3.2 Post-buprenorphine data collection

One day following baseline data collection, mice were weighed and administered 0.05 mg/kg buprenorphine (Vetergesic’, Reckitt-Coleman, Hull, UK) subcutaneously into the scruff of the animal. Forty-five minutes later, the process of collecting images of their face and video recording of their behaviour (described above), was repeated in order to determine if buprenorphine (BUP) alone resulted in any changes in MGS or behaviour. This dose of buprenorphine was used as it represents a recommended dose of analgesia of for mice following a surgical procedure (Flecknell, 2009).

#### 2.3.3 Post-isoflurane data collection

Forty-eight hours following the buprenorphine data collection, anaesthetic only control data were collected. Anaesthesia was induced in a perspex anaesthetic induction chamber (VetTech Solutions Ltd, Cheshire, UK) with isoflurane in oxygen (induction 5%, 2 L/min). Anaesthesia was then maintained (2.5%, 1.0 L/min) for 10 min to represent the duration of abdominal surgery (Leach et al., 2012). Mice were then allowed to recover. During the recovery phase, mice were monitored until able to walk around the cage. Thirty minutes following recovery, the process of collecting images of their faces and video recording of their behaviour (described above), was repeated in order to determine if isoflurane anaesthesia alone (ISO) resulted in any changes in MGS or behaviour.

Following a surgical procedure, mice are most intensely monitored during and immediately following recovery from anaesthesia. In this study, we opted to focus our assessment during the time frame equivalent to this phase.

Although data were collected in the same order for each mouse (i.e. baseline, BUP then ISO), it has been previously demonstrated that there is no change in MGS score when control mice are placed into the filming boxes on three separate occasions (Miller and Leach, 2015).

No adverse or unexpected events occurred following the administration of either isoflurane or buprenorphine. Data were collected from every animal at each stage of the study and all data were used in statistical analysis.

### 2.4 Data collection

#### 2.4.1 Behaviour

A 6-min epoch of video sequence of each mouse at each time point (baseline, post-buprenorphine, post-isoflurane anaesthesia) was analysed manually with ‘Cowlog 2.11’ (Hanninen and Pastell, 2009) using the ethogram shown in Table 1. Analysis was limited to only 6 min due to the extremely time consuming nature of manual scoring of mouse behaviour.

#### 2.4.2 MGS

The HD close up filming was viewed and screen shots were taken on every occasion that a clear image of the mouse's face was visible with the exception of when the mouse was grooming. These images were then cropped, leaving only the face of the mouse in the image. Using a random number generator (www.Random.org), one image per mouse, per time point was selected. Using the random sequence generator, the selected images were re-ordered and inserted into a custom designed Microsoft Excel file. Observers who were blinded to the experimental details, design and purpose scored each photograph using the five action units of the MGS as described by Langford et al. (2010). A MGS manual was provided to the scorers for training and reference, but the title of the manual was edited to ‘mouse facial action coding manual’ to limit biasing of scores from the title. Observers also received a short 10 min training session, detailing each of the action units. This has been the standard practice in our MGS studies conducted to date and has achieved high levels of both inter- and intra-observer reliability. Scores for each FAU for every individual photograph were then combined to produce a global MGS score for each image. As multiple individuals scored the images, the mean global score was then calculated.

### 2.5 Statistical analysis

Data were analysed using SPSS software. Behaviour data and MGS data were analysed non-parametrically. A Mann–Whitney *U* test compared the CBA to DBA/2 mice at each time point. A Friedman's test was used to compare MGS and behaviour scores over time, with post hoc analysis being carried out with Wilcoxon signed-rank test with a Bonferroni Correction for Multiple Comparisons being applied. Results were considered statistically significant when *p* < 0.05.

## 3 Results

### 3.1 Mouse grimace scale

Due to the high number of failed attempts to score the whiskers, this facial action unit was excluded from analysis. Maximum MGS score was therefore 8 rather than 10. No significant difference was found between CBA and DBA/2 mice at any individual time points throughout the study.

**Fig. 1:**
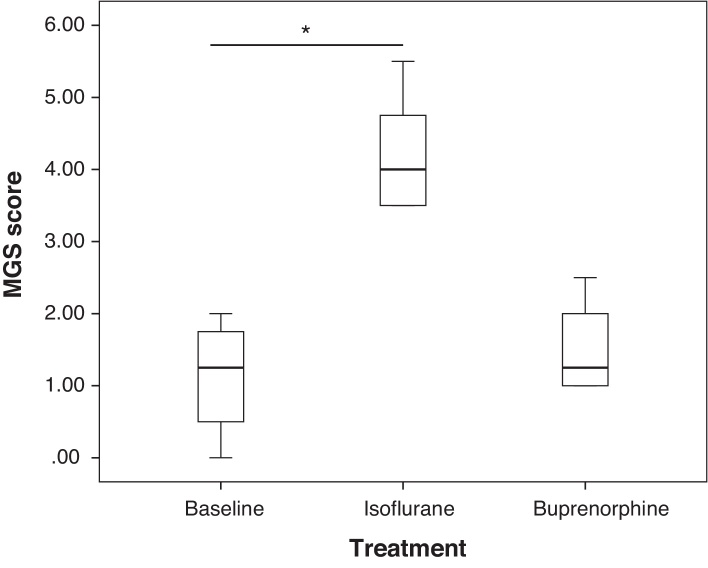
Box plot of MGS scores in DBA/2 mice at baseline and following either isoflurane anaesthesia or 0.05 mg/kg buprenorphine alone. The whisker FAU was excluded from the analysis (maximum obtainable score was 8).

In CBA mice, there was no significant difference in MGS scores between baseline compared to either post isoflurane anaesthesia or the buprenorphine administration. In DBA/2 mice, there was no significant difference in MGS scores at baseline compared to post buprenorphine administration. However, the MGS score following isoflurane anaesthesia was significantly higher than at baseline (*p* < 0.05) (Fig. 1).

### 3.2 Behaviour

A composite ‘pain behaviours’ group was included as indicated in Table 1. Behaviours in this category were those previously identified to occur following potentially painful procedure in mice and occur extremely infrequently in control/baseline animals (Wright-Williams et al., 2007, Miller et al., 2012, Leach et al., 2012).

**Fig. 2:**
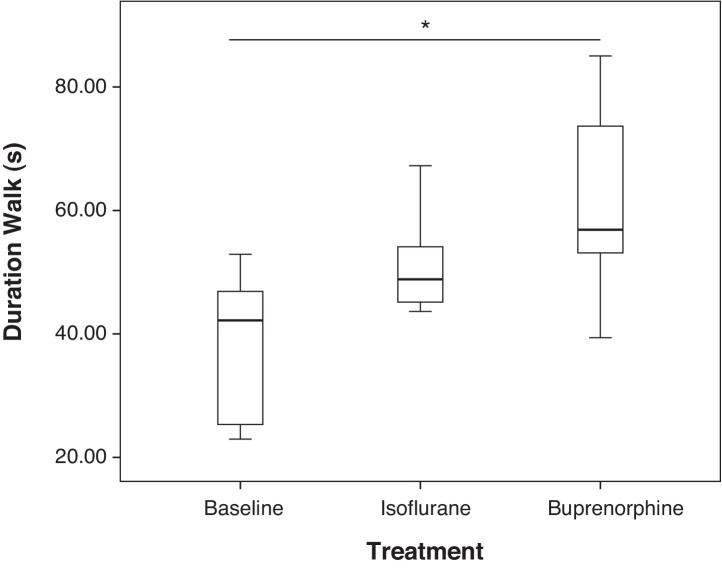
Box plots of duration of walking during 6 min of analysis, in CBA mice at baseline and following either isoflurane anaesthesia or 0.05 mg/kg buprenorphine alone.

Significant changes in the duration of time spent displaying certain behaviours were found in the ISO and BUP groups compared to baseline (Table 2 for full list of changes and associated *p* values). Changes in these behaviours were only identified in the CBA mice and not the DBA/2 mice. Most notably, CBA mice spent significantly longer walking (Fig. 2) and chewing bedding following a dose of buprenorphine compared to baseline (*p* < 0.05 for both comparisons).

## 4 Discussion

Behavioural analysis is a key method used in assessment of welfare (Sneddon et al., 2014). Although behavioural-based assessment represents an effective means of assessing pain in laboratory mice (Wright-Williams et al., 2007, Miller et al., 2012, Leach et al., 2012), its major limitation is the time taken to develop these indices and then to train observers to use them effectively (Roughan and Flecknell, 2006). Due to the expanding use of mice in biomedical research and the time consuming nature of developing relevant ethogram for new procedures and strains, novel rapid methods of assessment should be investigated and validated for use. The MGS has previously shown promise in for the effective assessment of pain in mice (Langford et al., 2010, Leach et al., 2012), but requires further validation before it can be widely implemented in clinical scenarios. In this study, we have investigated the influence of isoflurane anaesthesia and buprenorphine analgesia in non-painful mice to establish whether these procedures influence exhibition of pain behaviours and the MGS in two common mouse strains. Understanding whether these non-painful procedures, that are often integral to research procedures, have any effect on our assessment methods is critical to their further validation.

Limited changes in spontaneous behaviour were observed between before and after isoflurane anaesthesia or buprenorphine administration. Key, previously validated, pain associated behaviours such as belly pressing, twitching, flinching and writhing were not influenced by the administration of anaesthesia or buprenorphine alone. These subtle behaviours are vitally important in pain assessment of mice. They do not occur at baseline and their presence is noted following various routine laboratory procedures when analgesic provision is inadequate (Wright-Williams et al., 2007, Wright-Williams et al., 2013, Kawaura et al., 2011). The changes in behaviour following anaesthesia and analgesia that were demonstrated here were in line with previous studies which noted administration of buprenorphine increases activity (e.g. walking and hopping) in non-painful mice (Cowan et al., 1977, Tubbs et al., 2011, Wright-Williams et al., 2013). The increase in duration of time spent chewing bedding following buprenorphine administration, may represent pica behaviour which is thought to be a sign of nausea and has previously been associated with the administration of buprenorphine in rat (Clark et al., 1997). There is some evidence that mice also display pica behaviour (Yamamoto et al., 2002) although it is thought to be highly strain dependent and a less robust measure than in the rat (Stern et al., 2011).

Blind scoring with the mouse grimace scale revealed no change in scores between baseline and following administration of either isoflurane anaesthesia or buprenorphine in CBA mice. However, isoflurane did increase the MGS score compared to baseline in DBA/2 mice. This is an important finding and should be considered when attempting to score pain in this strain of mouse using the MGS if isoflurane anaesthesia is included in the research protocol, particularly when no baseline data are available for each animal. Like many published studies, this finding again highlights the importance of being familiar with the specific strain of mice being studied, as there is increasing evidence of considerable variation between strains in terms of their reactions to various procedures. In contrast to the specific pain behaviours, which either do not occur, or occur at a very low level when a painful stimulus is not present, the mouse grimace scale score at baseline is not zero. This is also reported in other studies (Leach et al., 2012, Miller and Leach, 2015), and must be considered when assessing mice using the MGS, particularly if baseline MGS scores are not available.

## 5 Conclusion

Behaviour is commonly used for assessing pain in mice with many specific pain associated behaviours having been previously identified (e.g. Wright-Williams et al., 2007). To effectively use these behavioural indices, we must be confident that their presence, in a given strain, is linked to pain and not another effect of the procedure. Here we demonstrated that isoflurane anaesthesia or a single dose of buprenorphine did not result in the presence of any specific previously identified, pain associated behaviours in CBA or DBA/2 mice.

A single 0.05 mg/kg s.c. injection had no effect on mouse grimace scale score in either DBA/2 or CBA male mice. Isoflurane anaesthesia of 10 min duration results in an increase in MGS score in DBA/2 mice and this effect should be considered when attempting to use the MGS in pain assessment following procedure requiring isoflurane anaesthesia (e.g. surgery) in this strain of mouse. Further work should be carried out to establish the presence of this isoflurane effect in other strains and the influence of gender to increase the validity of this method for pain assessment.

## Conflict of interest

All authors declare that they have no conflict of interests.

## Figures and Tables

**Table 1:** Behaviours scored and analysed during this study. Behaviours previously identified as being pain associated behaviours were grouped together during analysis to produce a composite ‘pain behaviour score’. The behaviours included in this composite score are indicated in column 3.

Behaviour	Definition	Pain behaviour group?
Climb	Vertical movement up cage sides	No
Hop	Hopping movement across the cage floor	No
Walk	Movement across cage floor using all 4 limbs	No
Chew bedding	Gnawing sawdust bedding	No
Rearing	Standing on rear legs, full or partial stretch	No
Groom	Grooming of the body, head, limbs or tail	No
Inactive	No movement around the cage	No
Stagger	Partial loss of balance when walking	Yes
Raised tail	When walking, tail is raised from the floor	Yes
Rear leg lift	One rear leg is lifted briefly straight out behind the body	Yes
Shake	Rapid side to side movement of the body	Yes
Twitch	Rapid contraction of the back muscles	Yes
Writhe	Contortion of abdominal muscles	Yes
Flinch	Small movement involving whole body	Yes
Belly press	Pressing of abdomen toward cage floor	Yes

**Table 2:** Significant changes in duration of time spent displaying behaviours between baseline and isoflurane (ISO) or buprenorphine (BUP) groups. NS, no significant difference, number given is *p* value.

Behaviour	CBA mice	DBA/2 mice
Walk	Base < BUP 0.025	NS
Hop	Base < BUP 0.017	NS
Climb	Base > BUP 0.012	NS
Inactive	Base < BUP 0.012	NS
Chew bedding	Base < BUP 0.017	NS
Rearing	Base > BUP 0.012	NS
